# Preoperative lymph-node enlargement is associated with favorable survival for patients with pathological stage II colorectal cancer

**DOI:** 10.1007/s00595-026-03440-8

**Published:** 2026-08-12

**Authors:** Emi Ota, Mayumi Ozawa, Shigeru Yamagishi, Kazuya Nakagawa, Koichi Mori, Kanechika Den, Hirokazu Suwa, Itaru Endo

**Affiliations:** 1 Department of Surgery, Fujisawa City Hospital, Fujisawa, Japan; 2 Department of Surgery, Gastroenterological Center, Yokohama City University Medical Center, 4-57, Urafune-cho, Minami-ku, Yokohama-shi, 232-0024 Japan; 3 Department of Gastroenterological Surgery, Yokohama City University Graduate school of Medicine, Yokohama, Japan; 4 Department of Surgery, Yokosuka Kyosai Hospital, Yokosuka, Japan

**Keywords:** Colorectal cancer, Pathological stage II, Preoperative lymph node enlargement, Overall survival, Relapse-free survival

## Abstract

**Purpose:**

The importance of preoperative lymph-node enlargement as a prognostic factor for patients with colorectal cancer remains unclear because of inconsistent staging and size criteria. We conducted this study to evaluate its clinical impact, using a standardized definition for pathological stage II colorectal cancer.

**Methods:**

The subjects of this retrospective study were patients with pathological stage II colorectal adenocarcinoma, who underwent curative resection between January 2009 and December 2020. Patients were categorized based on preoperative lymph-node enlargement, defined as a short-axis diameter ≥5 mm on imaging. Independent risk factors for 5-year overall survival (OS) and relapse-free survival (RFS) were investigated.

**Results:**

A total of 562 patients were included (185 without and 377 with enlarged lymph nodes), and the median follow-up period was 61.8 months. Multivariate analysis identified that preoperative hemoglobin level < 12 g/dL (hazard ratios (HRs) = 1.937, 95% confidence interval (CI) = 1.181–3.175, *p* = 0.009) and preoperative bowel obstruction (hazard ratios (HRs) = 2.060, 95% confidence interval (CI) = 1.066–3.979, *p* = 0.031) were independent factors for poor OS. Conversely, lymph node enlargement was independently associated with favorable OS (HRs = 0.503, 95% CI, 0.316–0.801, *p* = 0.004). Pathological T4 stage was an independent factor for poor RFS (HRs = 2.973, 95% CI = 1.752–5.044, *p* < 0.001).

**Conclusions:**

Preoperative lymph-node enlargement predicts favorable OS for patients with pathological stage II colorectal cancer.

**Supplementary Information:**

The online version contains supplementary material available at https://doi.org/10.1007/s00595-026-03440-8.

## Introduction

The presence of lymph node metastasis in patients with colorectal cancer defines pathological stage III disease and is associated with higher recurrence rates and poorer prognosis [1–3]. Conversely, in clinical practice, a subset of patients who are diagnosed preoperatively with clinical stage III disease based on radiologically suspected lymph-node metastasis, are ultimately classified as having pathological stage II disease following postoperative histopathological examination.

The lymph nodes of the colon and rectum function not only as a drainage system for interstitial fluid, but also as essential immunological structures. During the inflammatory process, the proliferation of lymphocytes and mesenchymal tissue is commonly observed [4, 5]. The host immune response within tumor tissues has been shown to be associated with improved survival outcomes for patients with various types of cancer [6–8]. In colorectal cancer, a high density of inflammatory lymphocytes has been associated with favorable clinical outcomes [9–11]. Conversely, Song et al. reported that lymph-node enlargement is associated with a poor prognosis [12]. However, previous reports were limited by heterogeneous disease stages and many were based primarily on pathological findings. Although Matsunaga et al. investigated the relationship between preoperative lymph-node enlargement, assessed clinically, and prognosis, the criteria for defining lymph-node enlargement have not been standardized [11]. Consequently, the association between consistently defined lymph-node enlargement and true clinical outcomes remains unclear.

In the present study, we focused on patients ultimately diagnosed with pathological stage II colorectal cancer and investigated the association between preoperative lymph-node enlargement assessed clinically, and prognosis, using standardization criteria for lymph-node enlargement.

## Methods

### Patient selection

This multicenter retrospective observational study was conducted at Fujisawa City Hospital and Yokohama City University Hospital. The study was approved by our institutional review board (No. B180400017) and conducted in accordance with the principles of the Declaration of Helsinki. Informed consent was obtained from all the patients. The study adhered to the STROBE (Strengthening the Reporting of Observational Studies in Epidemiology) statement.

Patients who underwent colorectal cancer surgery between January 2009 and December 2020 were enrolled in the present study. Patients were consecutively selected from the database to reduce selection bias. The inclusion criteria were as follows: primary colorectal adenocarcinoma, pathological stage II disease, and complete resection of colorectal cancer. Patients with diseases other than primary tumors such as tumor recurrence and diverticula, neoadjuvant chemotherapy or radiotherapy, postoperative 30-day mortality (defined as death within 30 days of surgery), or incomplete data, were excluded from this study. For patients with synchronous double colorectal cancers, the more advanced lesion was selected as the target lesion for analysis.

### Definition of swollen lymph nodes

Previous studies have reported that large lymph nodes are often not associated with metastatic disease but instead reflect lymphoid follicular hyperplasia [13–15]. Markle et al. reported that the positivity rate of lymph nodes with a short-axis diameter ≥ of 10 mm was only 28%, indicating that 72% of lymph nodes were negative, whereas the majority of metastatic lymph nodes (73%) had a diameter of ˂ 10 mm [16]. Recent studies have described the application of a size criterion of a 5 mm maximum short-axis nodal diameter for distinguishing between benign and malignant nodes, but at best, this has moderate sensitivity and specificity for the detection of nodal metastases [17–19]. A recent meta-analysis of imaging studies found no significant differences between computed tomography (CT) and magnetic resonance imaging (MRI) for nodal staging [20]. Many of these studies adopted the size criteria of 5 mm for distinguishing between malignant and benign nodes. CT imaging was found to have a sensitivity of 55% and a specificity of 74%, while MRI was found to have a sensitivity of 66% and a specificity of 76%. A recent study involving 694 patients with colon cancer reported that the optimal cutoff values for predicting lymph node metastasis on preoperative CT were 6.5 mm for the long-axis diameter and 5.5 mm for the short-axis diameter [21]. Based on these findings, we defined clinically positive lymph nodes as those with a short-axis diameter of ≥ 5 mm on preoperative imaging. Lymph nodes were assessed using CT scan for colon cancer and MRI for rectal cancer. The images were evaluated by one attending outpatient physician and one operating surgeon, and the final assessment was confirmed at a conference with at least 10 participants. As this study focused on patients with pathological stage II colorectal cancer, patients with suspected distant lymph-node metastasis corresponding to stage IV disease were excluded. Thus, the present analysis evaluated the presence or absence of enlargement of the regional lymph nodes.

### Observed indicators

Clinical and demographic information was obtained to provide a comprehensive understanding of the patient population. This included age, sex, body mass index (BMI), American Society of Anesthesiologist Physical Status Classification System (ASA-PS), allergy, tumor location, preoperative bowel obstruction or perforation, and preoperative laboratory data, including blood count and biochemistry. Operative data included the surgical approach, procedure, operative time, and blood loss. Pathological data included tumor differentiation, pathological T stage, the number of lymph nodes examined, and lymphatic and venous invasion. Clinical and pathological staging were determined using the 8th edition of the TNM classification by the Union for International Cancer Control (UICC) [22]. Postoperative data included adjuvant chemotherapy, median follow-up period, 5-year relapse-free survival (RFS), and 5-year overall survival (OS).

### Statistical analysis

All statistical analyses were performed using EZR (ver. 3.0; Saitama Medical Center, Jichi Medical University, Saitama, Japan). Continuous variables were reported as medians. Categorical variables (compared using Fisher’s exact test or the chi-squared test) and continuous variables (compared using the Mann–Whitney U test) were analyzed. The 5-year RFS, the 5-year OS, and the 5-year cancer-specific survival rates were calculated using the Kaplan–Meier method, and the factors affecting RFS and OS were analyzed using univariate and multivariate analyses. Univariate analyses were performed using the log-rank test, and multivariate analyses were conducted using a Cox proportional hazards model. P values of < 0.05 were considered significant. This time-to-event analysis provided hazard ratios (HRs) with 95% confidence intervals (CIs).

## Results

### Patient background

Table 1 summarizes the patient demographics and the parameters of the non-enlarged and enlarged lymph nodes. A total of 562 patients from two institutions were analyzed, including 309 (55.0%) men and 253 (45.0%) women. There were 185 patients with non-enlarged lymph nodes, and 377 with enlarged lymph nodes. There were no significant differences in sex, BMI, smoking, ASA-PS, allergy, tumor location, or preoperative bowel obstruction or perforation between the groups; however, the patients in the non-enlarged lymph-node group were older (73 vs. 72 years, *p* = 0.030). Preoperative laboratory data demonstrated lower hemoglobin (11.8 g/dL vs. 12.6 g/dL, p˂0.001) and albumin (3.9 g/dL vs. 4.1 g/dL, p˂0.001) levels and a higher C-reactive protein (CRP) level (0.17 mg/dL vs. 0.09 mg/dL, p˂0.001) in the enlarged lymph-node group. There were no differences in white blood cell (WBC) count or hemoglobin A1c (HbA1c), CEA, or CA19-9 levels between the groups.

**Table 1:** Clinical characteristics of the patients in the non-enlarged lymph-node and the enlarged lymph-node groups

	*All (n = 562)*	*Non-enlarged lymph node (n = 185)*	*Enlarged lymph node (n = 377)*	*p*
Age, median (IQR), years	72 (65–79)	73 (66–81)	72 (65–78)	0.030
Sex				0.367
Male	309 (55.0%)	107 (57.8%)	202 (53.6%)	
Female	253 (45.0%)	78 (42.2%)	175 (46.4%)	
BMI, median (IQR), kg/m^2^	22.0 (20.0–24.5)	21.9 (19.8–24.5)	22.0 (20.1–24.3)	0.578
Smoking				0.648
No	331 (58.9%)	106 (57.30%)	225 (59.7%)	
Yes	231 (41.1%)	79 (42.7%)	152 (40.3%)	
ASA-PS score				0.156
I	70 (12.5%)	16 (8.7%)	54 (14.3%)	
II	430 (76.5%)	148 (80.0%)	282 (74.8%)	
III	62 (11.0%)	21 (11.3%)	41 (10.9%)	
IV	0 (0%)	0 (0%)	0 (0%)	
Allergy				0.247
No	541 (96.3%)	175 (94.6%)	366 (97.1%)	
Yes	21 (3.7%)	10 (5.4%)	11 (2.9%)	
Location				0.189
Right-sided colon	235 (41.8%)	69 (37.3%)	166 (44.0%)	
Left-sided colon	188 (33.5%)	71 (38.4%)	117 (31.0%)	
Rectum	139 (24.7%)	45 (24.3%)	94 (24.9%)	
Preoperative bowel obstruction				0.85
No	547 (97.3%)	181 (97.8%)	366 (97.1%)	
Yes	15 (2.7%)	4 (2.2%)	11 (2.9%)	
Preoperative bowel perforation				0.783
No	516 (91.8%)	172 (93.0%)	344 (91.2%)	
Yes	46 (8.2%)	13 (7.0%)	33 (8.8%)	
Preoperative WBC level, median (IQR), μ/L	6200 (5200–7500)	6100 (5100–7500)	6300 (5300–7425)	0.338
Preoperative hemoglobin level, median (IQR), g/dL	12.2 (10.4–13)	12.6 (11.1–13.7)	11.8 (10.1–13.2)	<0.001
Preoperative albumin level, median (IQR), g/dL	4.0 (3.6–4.3)	4.1 (3.7–4.4)	3.9 (3.6–4.2)	<0.001
Preoperative HbA1c level, median (IQR), mmol/mol	5.8 (5.5–6.2)	5.7 (5.4–6.2)	5.8 (5.5–6.1)	0.386
Preoperative CRP level, median (IQR), mg/dL	0.13 (0.05–0.53)	0.09 (0.04–0.30)	0.17 (0.06–0.71)	<0.001
Preoperative CEA level, median (IQR), ng/dL	3.5 (2.15–6.6)	3.4 (2.2–6.1)	3.8 (2.1–7.4)	0.246
Preoperative CA19-9 level, median (IQR), U/mL	10 (5.8–18.9)	11.0 (6.0–17.3)	9.9 (5.5–19.0)	0.663
Approaches				0.072
Open surgery	98 (17.4%)	23 (12.4%)	75 (19.9%)	
Conventional laparoscopic surgery	451 (80.2%)	157 (84.9%)	294 (78.0%)	
Robotic-assisted laparoscopic surgery	13 (2.3%)	5 (2.7%)	8 (2.11%)	
Procedures				0.841
Colectomy	406 (72.2%)	135 (73.0%)	271 (71.9%)	
Rectal resection	156 (27.8%)	50 (27.0%)	106 (28.1%)	
Operative time, median (IQR), min	213 (170–271)	206 (171–260)	215 (170–274)	0.369
Blood loss, median (IQR), mL	2 (5–78)	10 (5–50)	14 (5–92.5)	0.158
Tumor diameter, median (IQR), mm	45 (35–60)	35 (26–45)	50 (40–65)	<0.001
Tumor differentiation				0.111
Well/mod	527 (93.8%)	179 (96.8%)	348 (92.3%)	
Por/sig/muc	33 (5.9%)	6 (3.2%)	27 (7.2%)	
Others	2 (0.4%)	0 (0%)	2 (0.5%)	
Pathological T stage				<0.001
T3	451 (80.2%)	165 (89.2%)	286 (75.9%)	
T4	111 (19.8%)	20 (10.8%)	91 (24.1%)	
Number of lymph nodes examined, median (IQR)	21 (13–27)	18 (12–26)	25 (19–34)	<0.001
Lymphatic invasion				0.929
Negative	285 (50.7%)	93 (50.3%)	192 (50.9%)	
Positive	277 (49.3%)	92 (49.7%)	185 (49.1%)	
Venous invasion				0.513
Negative	203 (36.1%)	63 (34.1%)	140 (37.1%)	
Positive	359 (63.9%)	122 (66.0%)	237 (62.9%)	
Adjuvant chemotherapy	87 (15.5%)	25 (13.5%)	62 (16.4%)	0.388
Recurrence rate	60 (10.7%)	19 (10.3%)	41 (10.9%)	0.855
Liver	26 (4.6%)	10 (5.4%)	16 (4.2%)	
Lung	15 (2.7%)	4 (2.2%)	11 (2.9%)	
Peritoneal dissemination	16 (2.8%)	2 (1.1%)	14 (3.7%)	
Local	6 (1.1%)	2 (1.1%)	4 (1.1%)	
Others	7 (1.2%)	4 (2.2%)	3 (0.8%)	
Median follow-up period (IQR), months	61.8 (44.3–76.6)	61.3 (40.0–73.1)	62.1 (48.1–78.0)	0.043

IQR, interquartile range; BMI, body mass index; ASA-PS, American Society of Anesthesiologists Physical Status Classification System; HTN, hypertension; WBC, white blood cell; HbA1c, Hemoglobin A1c; CRP, C-reactive protein

There were no significant differences in surgical approach, operative time, or blood loss between the groups. With respect to pathological factors, there were no significant differences in histological type or lymphatic or venous invasion. However, the enlarged lymph-node group had a significantly larger tumor size (50 mm vs. 35 mm, *p* < 0.001), a higher proportion of pathological T4 stage (24.1% vs. 10.8%, p˂0.001) and a significantly greater number of harvested lymph nodes (25 vs. 18, p˂0.001). No significant differences were observed between the two groups in the rate of adjuvant chemotherapy administration (16.4% vs. 13.5%, *p* = 0.388) or recurrence (10.9% vs. 10.3%, *p* = 0.855).

### Risk factors for 5-year OS

**Fig. 1:**
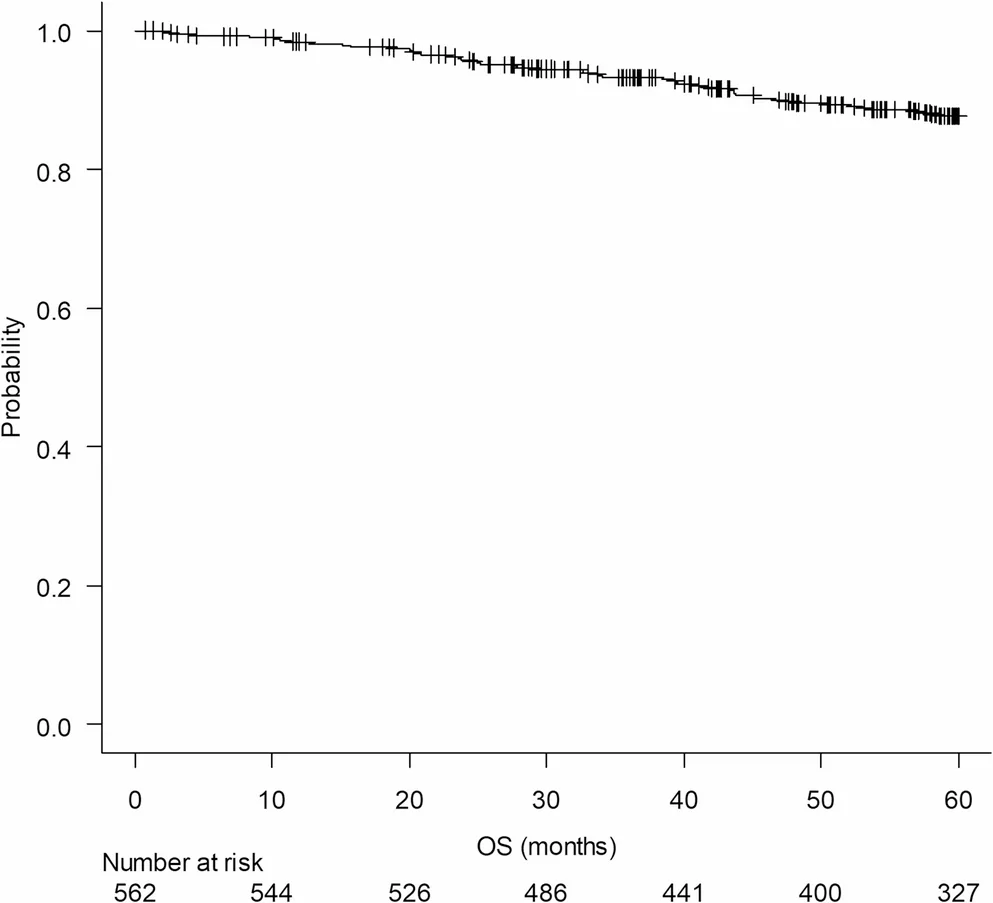
Kaplan–Meier survival curves for the 5‑year overall survival of all patients

**Fig. 2:**
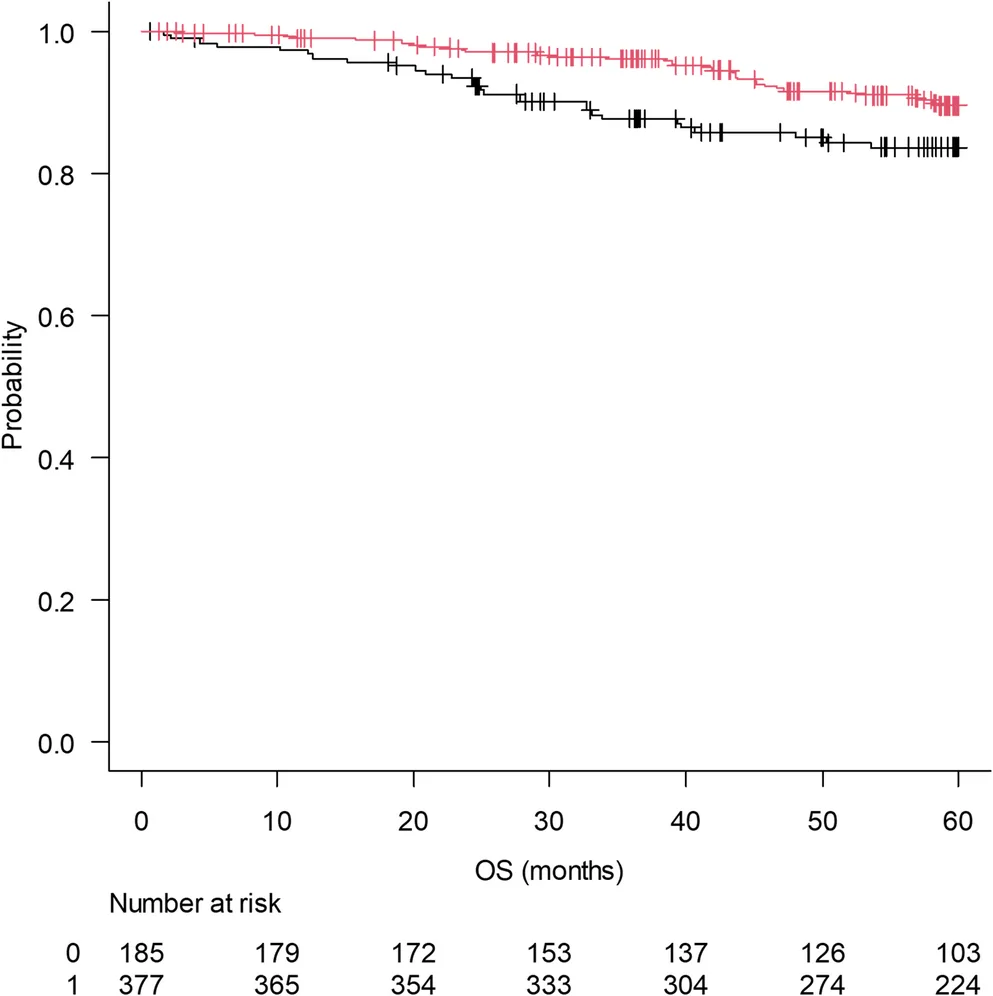
Kaplan–Meier survival curves for the 5‑year overall survival of patients with clinical N stage 0 and 1 or 2 colorectal cancer. The red line represents the preoperative enlarged lymph-node group and the black line represents the non-enlarged lymph-node group

**Fig. 3:**
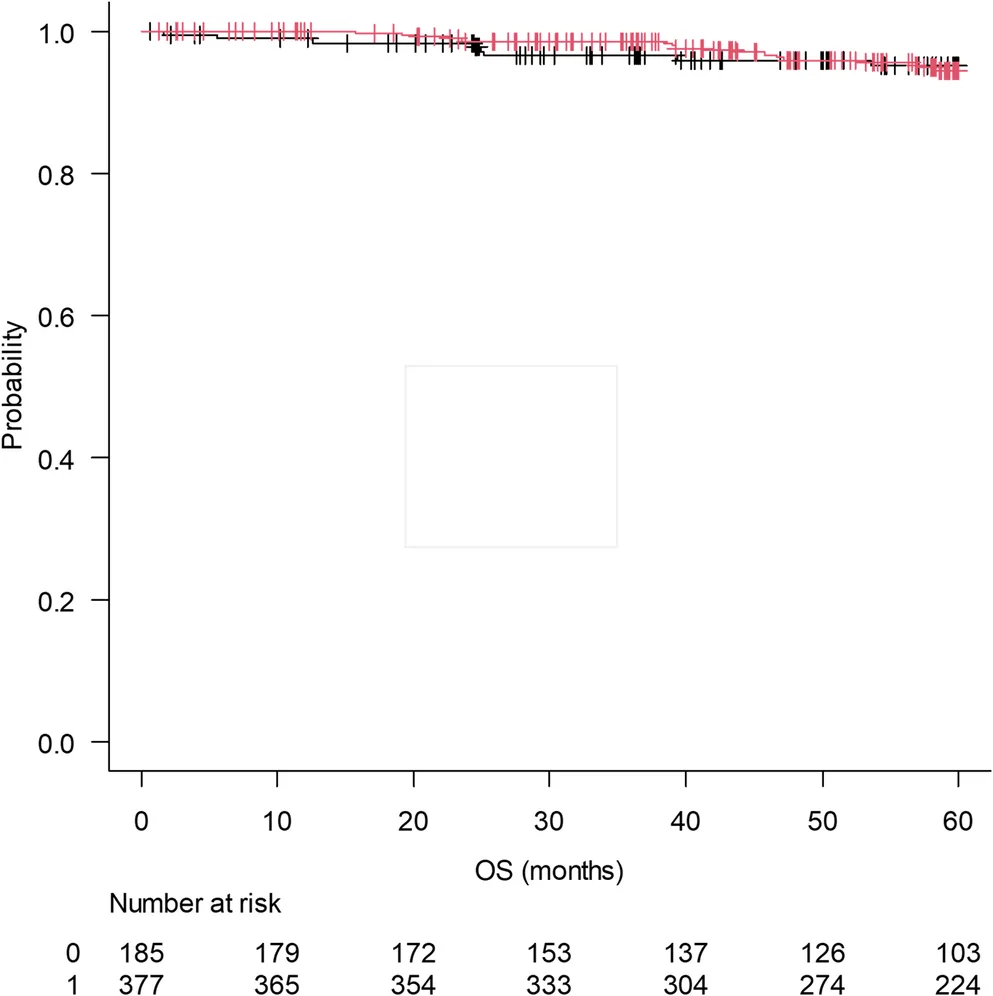
Kaplan–Meier survival curves for the 5‑year cancer-specific survival of patients with clinical N stage 0 and 1 or 2 colorectal cancer. The red line represents the preoperative enlarged lymph-node group and the black line represents the non-enlarged lymph-node group

Figure 1 shows the 5-year OS curve for all the patients. The 5-year OS rate was 87.6%. Table 2 shows the results of the univariate and multivariate analyses of 5-year OS. Univariate analyses revealed a significantly shorter OS for patients (cutoffs based on median values) ≥ 70 years of age, with preoperative hemoglobin level < 12 g/dL, preoperative albumin level < 4.0 g/dL, preoperative CRP level > 0.3 mg/dL, preoperative CEA level > 5.0 ng/dL, preoperative bowel obstruction, clinical N stage 0 (non-enlarged lymph nodes), pathological T4 stage, and number of harvested lymph nodes < 12. A multivariate analysis was performed for factors that showed significance (*p* < 0.05) in the univariate analysis. The multivariate analysis identified preoperative hemoglobin level < 12 g/dL (HRs = 1.937, 95% CI = 1.181–3.175, *p* = 0.009) and preoperative bowel obstruction (HRs = 2.060, 95% CI = 1.066–3.979, *p* = 0.031) as independent risk factors for a poor prognosis. In contrast, preoperative clinical N stage N1 or N2 (enlarged lymph nodes) was identified as an independent prognostic factor for favorable OS (HRs = 0.503, 95% CI, 0.316–0.801; *p* = 0.004). Figure 2 shows the survival curves of 5-year OS with clinical N stage 0 and 1 or 2 (83.6% vs. 89.5%, *p* = 0.027). Figure 3 shows the survival curves of 5-year cancer-specific survival with clinical N stage 0 and 1 or 2 (95.1% vs. 94.5%, *p* = 0.941). Table 3 compares the causes of death of patients in the non-enlarged lymph-node and enlarged lymph-node groups. Regarding the causes of death, infectious disease was approximately six times more frequent in the non-enlarged lymph-node group than in the enlarged lymph-node group (3.2% vs. 0.5%).

**Table 2:** Univariate and multivariate analyses of 5-year overall survival after resection of colon cancer

Variable	*All (n =562)*			Univariate analysis (log-rank) *p*-value	Multivariate analysis (Cox hazard)	
	No. of patients		5-year OS		HRs (95% CI)	*p*-value
Age, years				0.027	1.343 (0.823–2.191)	0.239
<70	211 (37.5%)		90.6%			
≥70	351 (62.5%)		85.8%			
Sex				0.256		
Male	309 (55.0%)		86.2%			
Female	253 (45.0%)		89.2%			
Preoperative WBC level, μ/L				0.575		
≤8500	503 (89.5%)		87.3%			
>8500	59 (10.5%)		89.6%			
Preoperative hemoglobin level, g/dL				<0.001	1.937 (1.181–3.175)	0.009
<12	263 (46.8%)		82.0%			
≥12	299 (53.2%)		92.3%			
Preoperative albumin level, g/dL				<0.001	1.587 (0.917–2.748)	0.099
<4.0	266 (47.3%)		81.3%			
≥4.0	296 (52.7%)		92.9%			
Preoperative CRP level, mg/dL				0.005	1.034 (0.622–1.718)	0.899
≤0.3	375 (66.7%)		90.2%			
>0.3	187 (33.3%)		82.2%			
Preoperative CEA level, ng/dL				0.014	1.372 (0.879–2.142)	0.164
≤5.0	371 (66.0%)		89.8%			
>5.0	191 (34.0%)		83.3%			
Tumor location				0.343		
Right sided colon	235 (41.8%)		84.0%			
Left sided colon	188 (33.5%)		90.4%			
Rectum	139 (24.7%)		89.8%			
Preoperative bowel obstruction				<0.001	2.060 (1.066–3.979)	0.031
No	529 (94.1%)		88.7%			
Yes	33 (5.9%)		70.5%			
Preoperative bowel perforation				0.177		0
No	547 (97.3%)		87.6%			
Yes	15 (2.7%)		78.6%			
Clinical-N stage				0.027	0.503 (0.316–0.801)	0.004
N0	184 (32.7%)		83.6%			
N1/2	378 (67.3%)		89.5%			
Tumor diameter, mm				0.123		
<45	249 (44.3%)	89.0%				
≥45	313 (55.7%)	86.4%				
Tumor differentiation				0.361		
Well/mod	526 (93.6%)		87.8%			
Por/sig/muc	33 (5.9%)		83.0%			
Others	3 (0.5%)		100%			
Depth of tumor invasion				0.041	1.560 (0.936–2.602)	0.088
Pathological T3	451 (80.2%)		88.8%			
Pathological T4	111 (19.8%)		82.7%			
Number of harvested lymph node				0.023	1.553 (0.932–2.587)	0.091
<12	106 (18.9%)		80.9%			
≥12	456 (81.1%)		89.1%			
Venous invasion				0.508		
Negative	203 (36.1%)		87.1%			
Positive	359 (63.9%)		87.8%			
Lymphatic invasion				0.071		
Negative	284 (50.5%)		89.5%			
Positive	278 (49.5%)		85.6%			
Adjuvant chemotherapy				0.074		
No	474 (84.3%)		86.6%			
Yes	88 (15.7%)		92.6%			

WBC, white blood cell; CRP, C-reactive protein; HRs, hazard ratios; CI, confidence interval

**Table 3:** Causes of death of patients in the non-enlarged lymph-node and enlarged lymph-node groups

	*All (n = 562)*	*Non-enlarged lymph node (n = 185)*	*Enlarged lymph node (n = 377)*
Death from colorectal cancer	24 (4.3%)	7 (3.8%)	17 (4.5%)
Infectious disease	9 (1.6%)	6 (3.2%)	2 (0.5%)
Cardiovascular disease	10 (1.8%)	5 (2.7%)	5 (1.3%)
Cerebrovascular disease	4 (0.7%)	2 (1.1%)	2 (0.5%)
Liver failure	1 (0.2%)	1 (0.5%)	0 (0%)
Autoimmune disease	1 (0.2%)	0 (0%)	1 (0.3%)
Other malignancies	21 (3.7%)	7 (3.8%)	14 (3.7%)
Accident or suicide	2 (0.4%)	1 (0.5%)	1 (0.3%)
Unknown	10 (1.8%)	5 (2.7%)	5 (1.3%)

### Risk factors for 5-year RFS

**Fig. 4:**
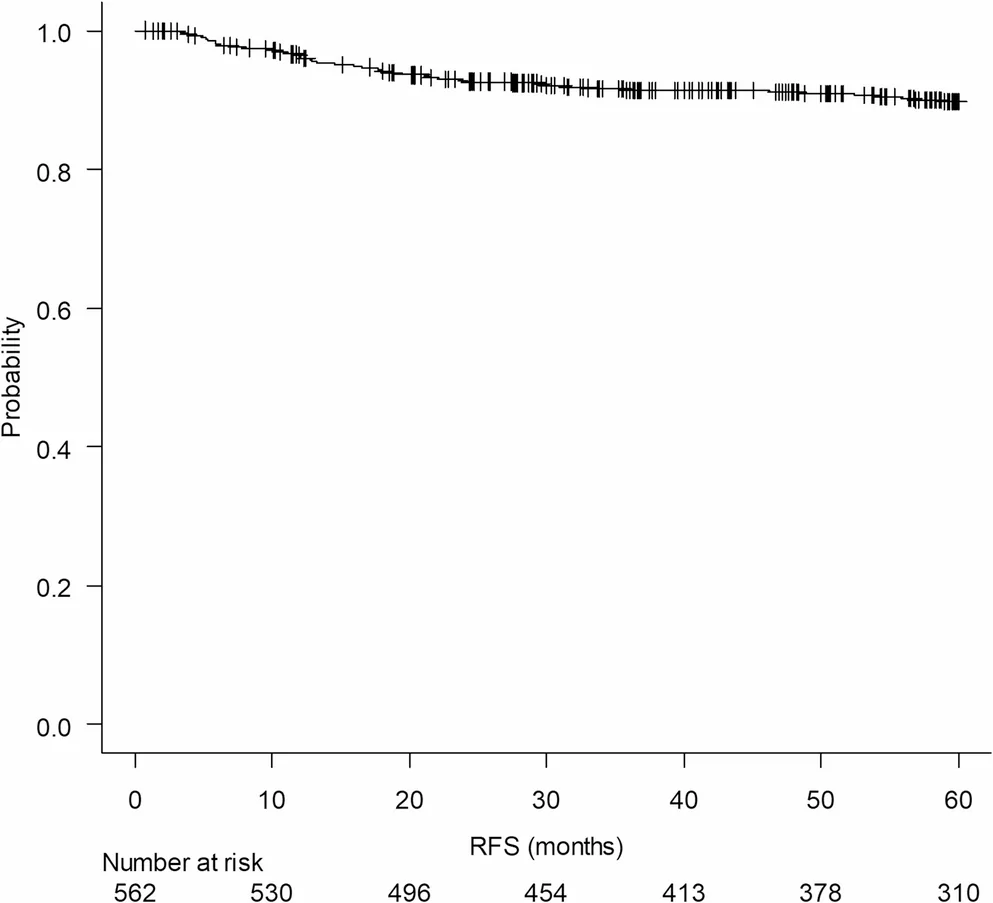
Kaplan–Meier survival curves for the 5‑year relapse-free survival of all patients

**Fig. 5:**
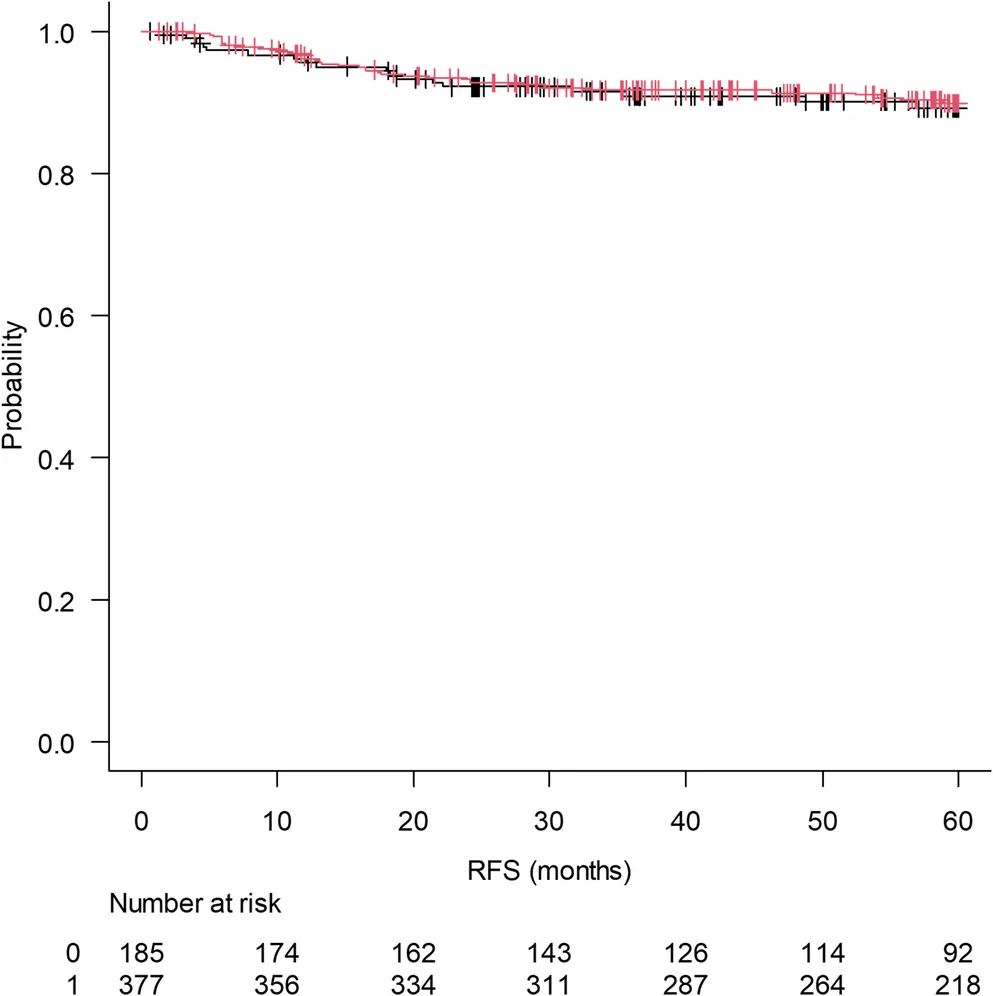
Kaplan–Meier survival curves for the 5‑year relapse-free survival of patients with clinical N stage 0 and 1 or 2 colorectal cancer. The red line represents the preoperative enlarged lymph-node group and the black line represents the non-enlarged lymph-node group

Figure 4 shows the 5-year RFS survival curves for all patients. The 5-year RFS rate was 89.7%. Table 4 shows the univariate and multivariate analyses of 5-year RFS. The univariate analysis showed significantly shorter RFS of patients with a preoperative albumin level < 4.0 g/dL, preoperative bowel obstruction and pathological T4 stage disease. A multivariate analysis was performed of the factors that showed significance (*p* < 0.05) in the univariate analysis. Pathological T4 stage was identified as an independent risk factor for poor RFS (HRs = 2.973, 95% CI = 1.752–5.044, *p* < 0.001). Figure 5 shows the survival curves of 5-year RFS of patients with clinical N stages 0 and 1 or 2 disease (89.2% vs. 89.9%, *p* = 0.943).

**Table 4:** Univariate and multivariate analyses of 5-year relapse free survival after resection of colon cancer

Variable	*All (n =562)*			Univariate analysis (log-rank) *p*-value	Multivariate analysis (Cox hazard)	
	No. of patients		5-year RFS		HRs (95% CI)	*p*-value
Age, years				0.669		
<70	211 (37.5%)		88.6%			
≥70	351 (62.5%)		90.4%			
Sex				0.876		
Male	309 (55.0%)		89.3%			
Female	253 (45.0%)		90.0%			
Preoperative WBC level, μ/L				0.858		
≤8500	503 (89.5%)		89.5%			
>8500	59 (10.5%)		90.6%			
Preoperative hemoglobin level, g/dL				0.328		
<12	263 (46.8%)		88.6%			
≥12	299 (53.2%)		90.6%			
Preoperative albumin level, g/dL				0.027	1.322 (0.768–2.275)	0.314
<4.0	266 (47.3%)		85.9%			
≥4.0	296 (52.7%)		92.8%			
Preoperative CRP level, mg/dL				0.218		
≤0.3	376 (66.9%)		91.3%			
>0.3	186 (33.1%)		86.1%			
Preoperative CEA level, ng/dL				0.273		
≤5.0	371 (66.0%)		90.8%			
>5.0	191 (34.0%)		87.4%			
Tumor location				0.804		
Right sided colon	235 (41.8%)		89.8%			
Left sided colon	188 (33.5%)		89.4%			
Rectum	139 (24.7%)		89.8%			
Preoperative bowel obstruction				0.024	1.923 (0.851–4.344)	0.116
No	529		90.5%			
Yes	33		75.3%			
Preoperative bowel perforation				0.213		
No	547 (%)		89.8%			
Yes	15 (%)		84.6%			
Clinical N stage				0.943		
N0	185 (32.9%)		89.2%			
N1/2	377 (67.1%)		89.9%			
Tumor diameter, mm				0.085		
<45	250 (44.5%)	91.9%				
≥45	312 (55.5%)	87.8%				
Tumor differentiation				0.593		
Well/mod	527 (93.8%)		89.8%			
Por/sig/muc	33 (5.9%)		85.9%			
Others	2 (0.4%)		NA			
Depth of tumor invasion				<0.001	2.973 (1.752–5.044)	<0.001
Pathological T3	451 (80.2%)		93.2%			
Pathological T4	111 (19.8%)		75.8%			
Number of harvested lymph node				0.095		
<12	456 (81.1%)		90.8%			
≥12	106 (18.9%)		84.9%			
Venous invasion				0.777		
Negative	203 (36.1%)		88.3%			
Positive	359 (63.9%)		90.5%			
Lymphatic invasion				0.153		
Negative	285 (50.7%)		88.0%			
Positive	277 (49.3%)		91.5%			

### Analyses according to tumor location

We distinguished the cohorts by tumor location and analyzed data separately (Supplementary files). The analyses did not identify preoperative lymph node enlargement as an independent prognostic factor in either subgroup.

## Discussion

In the present study, preoperative enlargement of the regional lymph nodes was identified as an independent favorable prognostic factor for OS among all the patients with pathological stage II colorectal cancer, including those with pathological T3 and T4 disease, whereas a hemoglobin level < 12 g/dL was identified as an independent unfavorable prognostic factor for OS.

The regional lymph nodes are connected to the primary tumor through lymphatic vessels and are immunologically continuous with the tumor. Therefore, reactive enlargement of the regional lymph nodes may serve as an indicator of the host immune response against tumor cells [23]. Enlarged lymph nodes in patients with pathological N0 rectal cancer have been reported to contain a greater number of lymphoid follicles relative to non-enlarged lymph nodes [24]. The number and size of the lymph nodes have been reported to correlate with peritumoral inflammatory cell infiltration and may be associated with a favorable prognosis [23, 25, 26]. This is consistent with our finding in that the lymph-node enlargement group had a significantly higher number of retrieved lymph nodes. Matsunaga et al. reported that lymph-node enlargement was independently associated with better OS and recurrence-free survival of patients with stage II colorectal cancer limited to pathological T4b disease [11]. In contrast to our finding that lymph-node enlargement was uniformly defined as a short-axis diameter of ≥ 5 mm, in the multicenter analysis by Matsunaga et al., the criteria for clinical N-positive classification were not standardized. It has been suggested that the size criteria for lymph-node enlargement are not consistent across institutions, and that some centers incorporate additional parameters such as nodal shape, contrast enhancement, and nodal clustering, potentially contributing to heterogeneity in the definition of lymph-node enlargement [27, 28]. Märkle et al. reported that an association between lymph-node size and the OS of patients with stage I/II colorectal cancer was observed only in those with T3/T4 disease, whereas no significant association was found when those with T1/T2 disease were included in the analysis [16]. These findings suggest that such an association may exist in patients with stage II disease, whose tumors are relatively large. Märkle et al. also defined the cutoff value for lymph node enlargement as 5 mm and reported that a higher number of pathological lymph nodes measuring ≥ 5 mm was an independent predictor of a reduced risk of distant metastasis [16]. Huang et al. reported improved disease-free survival and OS in the reactive lymph-node group of patients with stage 0/I and II colorectal cancer [10]. However, as their analysis included stages 0 and I it was not conducted within a uniform stage. Relative to stage II tumors, stage 0 and I tumors are less likely to induce tumor-associated inflammation. Thus, to ensure a consistent biological background, we restricted our analysis to patients with stage II disease.

Although our preoperative lymph-node enlargement group showed significantly better OS, no significant differences were observed in RFS and cancer-specific survival. While the proportion of pathological T4 (an independent risk factor for RFS) was higher in the preoperative lymph-node enlargement group, RFS was comparable between the two groups. Moreover, a comparison of baseline characteristics revealed that patients in the preoperative lymph-node enlargement group had larger tumor size, more advanced anemia, higher CRP levels, and lower serum albumin levels, indicating a greater burden of advanced tumors. Despite these unfavorable features, RFS and cancer-specific survival were comparable between the groups, which may suggest that recurrence was relatively suppressed in the lymph-node enlargement group. This finding suggests that the activation of antitumor immune responses in more advanced pathological T4 tumors may have contributed to the improvement in RFS. It has been suggested that antitumor immune responses may be activated in tumors that invade adjacent organs [29]. Matsunaga et al. reported that pathological T4b tumors have a higher rate of distant metastasis than other pathological T categories, which may allow immunological effects to become more pronounced. Consequently, lymph-node enlargement was identified as an independent favorable prognostic factor for patients with pathological T4b stage II colorectal cancer [11]. Huang et al. reported that the reactive lymph-node enlargement group had significantly higher WBC counts and tended to have larger tumor sizes, which were considered to reflect inflammatory reactions within the lymph nodes. They suggested that benign enlarged lymph nodes indicate an immune response to tumor antigens [10].

On the other hand, the preoperative lymph-node enlargement group showed no significant differences in cancer-specific survival. This suggests that the observed difference in OS in this study may not reflect an antitumor effect, but rather the influence of non-cancer related mortality factors, such as frailty, comorbidities, nutritional status, or other systemic factors. Interestingly, in this study, deaths caused by infectious diseases were more than six times more frequent, and deaths caused by cardiovascular disease were approximately two times more frequent in the non-enlarged lymph-node group than in the enlarged lymph-node group. Immune responses have been reported to decline in elderly individuals. This decline is attributed to age-related degenerative changes in the lymph nodes, including fibrosis, fatty degeneration, and calcification, which predispose lymph nodes to morphological involution [30–32]. In our analysis, patients in the non-enlarged lymph node group were slightly older than those in the enlarged lymph node group. With age, lymph nodes become less likely to enlarge and decrease in number, which is considered to lead to a weakened immune response, not only against tumors but also against infections [30–32].

In the present analysis, the proportion of patients with pathological T4 disease was higher in the preoperative lymph-node enlargement group (24.1% vs. 10.8%, *p* < 0.001). Accordingly, the rate of peritoneal recurrence was also higher in this group (3.7% vs. 1.1%). Nagata et al. reported that metachronous peritoneal metastasis occurred in 16% of patients with pathological T4 stage I-III colorectal cancer and that pathological T4 was an independent risk factor for this [33]. In the present study, the rate of adjuvant chemotherapy did not differ between patients with and those without preoperative lymph-node enlargement. However, patients in the preoperative lymph-node enlargement group may have favorable OS related to non-cancer factors. Therefore, in colorectal cancer patients with pathological T4 (an independent risk factor for RFS), more proactive consideration of adjuvant chemotherapy may contribute to better postoperative outcomes.

Anemia is reported in up to 40% patients with colorectal cancer, probably caused by iron deficiency, systemic inflammation, and spontaneous tumor bleeding [34–36]. In addition to being an important marker of more advanced tumor stage and greater treatment intensity, anemia in colorectal cancer patients is a well-established risk factor for infection, impaired physical function, and poorer OS [37]. A systematic review and meta-analysis of eight studies demonstrated that preoperative anemia was significantly associated with worse OS [38]. Consistent with these findings, we identified preoperative hemoglobin levels < 12 g/dL as an independent prognostic factor for worse OS.

The strength of this study was that the analysis was limited to patients with pathological stage II colorectal cancer and lymph-node enlargement was evaluated using a clearly unified criterion of a short-axis diameter of ≥ 5 mm. However, this study had several limitations. First, it was a retrospective study. Second, although lymph-node enlargement was defined as a lymph-node diameter of ≥5 mm, this cutoff value may not be optimal. Moreover, detailed data on lymph-node size and the number of enlarged lymph nodes were not available for all patients, precluding additional analyses using alternative definitions or stratifications. Third, although age was not identified as an independent prognostic factor in the multivariate analysis, the possibility that age influenced the prognosis of the non-enlarged lymph-node group could not be excluded. Fourth, the lack of direct immunological and molecular biological data, such as MSI/MMR status, tumor-infiltrating lymphocytes, lymphoid follicular hyperplasia, and CD3/CD8-positive infiltration, limits whether preoperative lymph-node enlargement reflects antitumor immune activity. Fifth, previous studies have found substantial differences in lymph-node size and components of anti-tumor immunity between right- and left-sided colorectal tumors [21, 39]. However, in the present study, preoperative lymph-node enlargement was not identified as a significant factor in the location-specific analyses. Further studies with larger sample sizes are needed to clarify the clinical significance of lymph-node enlargement according to tumor location.

## Conclusion

The preoperative enlargement of regional lymph nodes in patients with stage II colorectal cancer is associated with better OS.

## Supplementary Information

Below is the link to the electronic supplementary material.

Supplementary Material 1
