# Supplementary material for: Preoperative lymph-node enlargement is associated with favorable survival for patients with pathological stage II colorectal cancer
Source: Surg Today. 2026 Aug 12;56(11):3079–89. doi: 10.1007/s00595-026-03440-8 (PMC13722766; doi:10.1007/s00595-026-03440-8)
Supplement: Supplementary file 1 [file 595_2026_3440_MOESM1_ESM.docx]

**SUPPLEMENTARY TABLE 1. Clinical characteristics of the patients with right-sided colon cancer in the non-enlarged lymph node and enlarged lymph node groups**

|  | *All (n = 235)* | *Non-enlarged lymph node group (n = 69)* | *Enlarged lymph node group (n = 166)* | *p* |
| --- | --- | --- | --- | --- |
| Age, median (IQR), years | 74 (68.5–80) | 77 (68–83) | 74 (69–79) | 0.165 |
| Sex |  |  |  | 0.886 |
| Male | 123 (52.3%) | 37 (53.6%) | 86 (51.8%) |  |
| Female | 112 (47.7%) | 32 (46.4%) | 80 (48.2%) |  |
| BMI, median (IQR), kg/m^2^ | 22.0 (19.8–24.3) | 21.8 (20.0–24.6) | 22.0 (19.8–24.2) | 0.853 |
| Smoking |  |  |  | 0.760 |
| No | 158 (67.2%) | 45 (65.2%) | 113 (68.1%) |  |
| Yes | 77 (32.8%) | 24 (34.8%) | 53 (31.9%) |  |
| ASA-PS score |  |  |  | 0.424 |
| I | 26 (11.1%) | 5 (7.2%) | 21 (12.7%) |  |
| II | 183 (77.9%) | 55 (79.7%) | 128 (77.1%) |  |
| III | 26 (11.1%) | 9 (13.0%) | 17 (10.2%) |  |
| IV | 0 (0%) | 0 (0%) | 0 (0%) |  |
| Allergy |  |  |  | 1 |
| No | 225 (95.7%) | 66 (95.7%) | 159 (95.8%) |  |
| Yes | 10 (4.3%) | 3 (4.3%) | 7 (4.2%) |  |
| Preoperative bowel obstruction |  |  |  | 0.797 |
| No | 216 (9.2%) | 63 (91.3%) | 153 (92.2%) |  |
| Yes | 19 (8.1%) | 6 (8.7%) | 13 (7.8%) |  |
| Preoperative bowel perforation |  |  |  | 0.363 |
| No | 229 (91.9%) | 66 (95.7%) | 163 (98.2%) |  |
| Yes | 6 (2.6%) | 3 (4.3%) | 3 (0.2%) |  |
| Preoperative WBC level, median (IQR), μ/L | 6300 (5300–7450) | 6300 (5200–7700) | 6300 (5300–7400) | 0.888 |
| Preoperative hemoglobin level, median (IQR), g/dL | 11.6 (9.8–12.8) | 12.1 (10.9–13.3) | 11.2 (9.6–12.6) | 0.005 |
| Preoperative albumin level, median (IQR), g/dL | 4.0 (3.6–4.2) | 3.9 (3.5–4.2) | 3.9 (3.5–4.1) | 0.013 |
| Preoperative HbA1c level, median (IQR), mmol/ mol | 5.7 (5.4–6.1) | 5.5 (5.3–6.0) | 5.7 (5.4–6.1) | 0.072 |
| Preoperative CRP level, median (IQR), mg/dL | 0.18 (0.06–0.98) | 0.10 (0.05–0.41) | 0.22 (0.07–1.33) | <0.001 |
| Preoperative CEA level, median (IQR), ng/dL | 3.0 (1.8–5.7) | 3.0 (1.9–4.8) | 2.9 (1.8–6.0) | 0.604 |
| Preoperative CA19-9 level, median (IQR), U/mL | 10 (5.9–18) | 10.8 (6.5–16) | 10.0 (5.1–18) | 0.585 |
| Approaches |  |  |  | 0.385 |
| Open surgery | 51 (21.7%) | 12 (17.4%) | 39 (23.5%) |  |
| Conventional laparoscopic surgery | 184 (78.3%) | 57 (82.6%) | 127 (76.5%) |  |
| Robotic-assisted laparoscopic surgery | 0 (0%) | 0 (0%) | 0 (0%) |  |
| Operative time, median (IQR), min | 194.5 (156–238.8) | 201 (161–257) | 186 (152–235) | 0.164 |
| Blood loss, median (IQR), mL | 12.5 (5–77.3) | 15 (5–75) | 12 (5–78) | 0.990 |
| Tumor diameter, median (IQR), mm | 45 (35–65) | 31 (25–48) | 50 (40–68.5) | <0.001 |
| Tumor differentiation |  |  |  | 0.304 |
| Well/mod | 210 (89.4%) | 65 (94.2%) | 145 (87.3%) |  |
| Por/sig/muc | 24 (10.2%) | 4 (5.8%) | 20 (12.0%) |  |
| Others | 1 (0.4%) | 0 (0%) | 1 (0.6%) |  |
| Pathological T stage |  |  |  | 0.028 |
| T3 | 190 (80.9%) | 62 (89.9%) | 128 (77.1%) |  |
| T4 | 45 (19.1%) | 7 (10.1%) | 38 (22.9%) |  |
| Number of lymph nodes examined, median (IQR) | 26 (21.3–34.5) | 22 (13–29) | 28 (20–37) | <0.001 |
| Lymphatic invasion |  |  |  | 0.774 |
| Negative | 108 (46.0%) | 33 (47.8%) | 75 (45.2%) |  |
| Positive | 127 (54.0%) | 36 (52.2%) | 91 (54.8%) |  |
| Venous invasion |  |  |  | 0.770 |
| Negative | 93 (39.6%) | 26 (47.7%) | 67 (40.4%) |  |
| Positive | 142 (60.4%) | 43 (62.3%) | 99 (59.6%) |  |
| Adjuvant chemotherapy | 35 (14.9%) | 11 (15.9%) | 24 (14.5%) | 0.841 |
| Recurrence rate | 23 (9.8%) | 3 (4.3%) | 20 (12.0%) | 0.408 |
| Liver | 8 (3.4%) | 1 (1.4%) | 7 (4.2%) |  |
| Lung | 6 (2.6%) | 0 (0%) | 6 (3.6%) |  |
| Peritoneal dissemination | 6 (2.6%) | 0 (0%) | 6 (3.6%) |  |
| Local | 3 (1.3%) | 1 (1.4%) | 2 (1.2%) |  |
| Others | 4 (1.7%) | 1 (1.4%) | 3 (1.8%) |  |
| Median follow-up period (IQR), months | 61.2 (43.2–75.1) | 61.3 (41.0–71.5) | 61.2 (44.3–76.7) | 0.421 |

IQR, interquartile range; BMI, body mass index; ASA-PS, American Society of Anesthesiologists Physical Status Classification System; HTN, hypertension; WBC, white blood cell; HbA1c, Hemoglobin A1c; CRP, C-reactive protein.

**SUPPLEMENTARY TABLE 2. Univariate** **and multivariate analyses of 5-year overall survival after resection of right-sided colon cancer**

| *Variable* | *All (n =235)* | |  | *Univariate analysis*  *(log-rank)* | *Multivariate analysis*  *(Cox hazard)* | |
| --- | --- | --- | --- | --- | --- | --- |
|  | *No. of patients* | | *5-year OS* | *p-value* | *HRs (95% CI)* | *p-value* |
| Age, years |  | |  | 0.667 |  |  |
| <70 | 70 (29.8%) | | 85.8% |  |  |  |
| ≥70 | 165 (70.2%) | | 83.3% |  |  |  |
| Sex |  | |  | 0.712 |  |  |
| Male | 123 (52.3%) | | 82.4% |  |  |  |
| Female | 112 (47.7%) | | 85.7% |  |  |  |
| Preoperative WBC level, μ/L |  | |  | 0.802 |  |  |
| ≤8500 | 216 (91.9%) | | 84.7% |  |  |  |
| >8500 | 19 (8.1%) | | 77.1% |  |  |  |
| Preoperative hemoglobin level, g/dL |  | |  | 0.098 |  |  |
| <12 | 135 (57.4%) | | 81.0% |  |  |  |
| ≥12 | 100 (42.6%) | | 87.8% |  |  |  |
| Preoperative albumin level, g/dL |  | |  | 0.003 | 1.908 (0.831–4.380) | 0.128 |
| <4.0 | 131 (55.7%) | | 77.2% |  |  |  |
| ≥4.0 | 104 (44.3%) | | 92.3% |  |  |  |
| Preoperative CRP level, mg/dL |  | |  | 0.009 | 1.210 (0.586–2.501) | 0.606 |
| ≤0.3 | 144 (61.3%) | | 89.0% |  |  |  |
| >0.3 | 91 (38.7%) | | 75.7% |  |  |  |
| Preoperative CEA level, ng/dL |  | |  | 0.004 | 1.951 (1.012–3.758) | 0.046 |
| ≤5.0 | 172 (73.2%) | | 88.5% |  |  |  |
| >5.0 | 63 (26.8%) | | 72.2% |  |  |  |
| Preoperative bowel obstruction |  | |  | 0.003 | 2.388 (1.039–5.490) | 0.040 |
| No | 216 (91.9%) | | 88.7% |  |  |  |
| Yes | 19 (8.1%) | | 70.5% |  |  |  |
| Preoperative bowel perforation |  | |  | 0.147 |  |  |
| No | 229 (97.4%) | | 84.5% |  |  |  |
| Yes | 6 (2.6%) | | 60.0% |  |  |  |
| Clinical-N stage |  | | | 0.663 |  |  |
| N0 | 69 (29.4%) | | 83.3% |  |  |  |
| N1/2 | 166 (70.6%) | | 84.3% |  |  |  |
| Tumor diameter, mm |  | | | 0.189 |  |  |
| <45 | 100 (42.6%) | 86.6% | |  |  |  |
| ≥45 | 135 (57.4%) | 81.9% | |  |  |  |
| Tumor differentiation |  | | | 0.566 |  |  |
| Well/mod | 210 (89.4%) | | 84.6% |  |  |  |
| Por/sig/muc | 24 (10.2%) | | 78.3% |  |  |  |
| Others | 1 (0.4%) | | NA |  |  |  |
| Depth of tumor invasion |  | | | 0.019 | 1.799 (0.897–3.607) | 0.098 |
| Pathological T3 | 190 (80.9%) | | 86.3% |  |  |  |
| Pathological T4 | 45 (19.1%) | | 74.5% |  |  |  |
| Number of harvested lymph node |  | | | 0.098 |  |  |
| <12 | 135 (57.4%) | | 81.0% |  |  |  |
| ≥12 | 100 (42.6%) | | 87.8% |  |  |  |
| Venous invasion |  | | | 0.924 |  |  |
| Negative | 93 (39.6%) | | 83.9% |  |  |  |
| Positive | 142 (60.4%) | | 83.6% |  |  |  |
| Lymphatic invasion |  | | | 0.461 |  |  |
| Negative | 108 (46.0%) | | 85.4% |  |  |  |
| Positive | 127 (54.0%) | | 82.8% |  |  |  |
| Adjuvant chemotherapy |  | |  | 0.024 |  |  |
| No | 200 (85.1%) | | 81.7% |  |  |  |
| Yes | 35 (14.9%) | | 96.7% |  |  |  |

WBC, white blood cell; CRP, C-reactive protein; HRs, hazard ratios; CI, confidence interval.

**SUPPLEMENTARY TABLE 3. Univariate and multivariate analyses of 5-year relapse-free survival after resection of right-sided colon cancer**

| *Variable* | *All (n =235)* | |  | *Univariate analysis*  *(log-rank)* | *Multivariate analysis*  *(Cox hazard)* | |
| --- | --- | --- | --- | --- | --- | --- |
|  | *No. of patients* | | *5-year RFS* | *p-value* | *HRs (95% CI)* | *p-value* |
| Age, years |  | |  | 0.364 |  |  |
| <70 | 70 (29.8%) | | 92.1% |  |  |  |
| ≥70 | 165 (70.2%) | | 89.0% |  |  |  |
| Sex |  | |  | 0.615 |  |  |
| Male | 123 (52.3%) | | 89.8% |  |  |  |
| Female | 112 (47.7%) | | 89.8% |  |  |  |
| Preoperative WBC level, μ/L |  | |  | 0.396 |  |  |
| ≤8500 | 216 (91.9%) | | 90.5% |  |  |  |
| >8500 | 19 (8.1%) | | 83.1% |  |  |  |
| Preoperative hemoglobin level, g/dL |  | |  | 0.692 |  |  |
| <12 | 135 (57.4%) | | 89.5% |  |  |  |
| ≥12 | 100 (42.6%) | | 90.3% |  |  |  |
| Preoperative albumin level, g/dL |  | |  | 0.015 | 2.093 (0.735–5.962) | 0.167 |
| <4.0 | 131 (55.7%) | | 85.5% |  |  |  |
| ≥4.0 | 104 (44.3%) | | 95.0% |  |  |  |
| Preoperative CRP level, mg/dL |  | |  | 0.120 |  |  |
| ≤0.3 | 144 (61.3%) | | 92.2% |  |  |  |
| >0.3 | 91 (38.7%) | | 85.7% |  |  |  |
| Preoperative CEA level, ng/dL |  | |  | 0.004 | 2.483 (1.0765–5.728) | 0.033 |
| ≤5.0 | 172 (73.2%) | | 93.7% |  |  |  |
| >5.0 | 63 (26.8%) | | 79.6% |  |  |  |
| Preoperative bowel obstruction |  | |  | 0.074 |  |  |
| No | 216 (91.9%) | | 91.0% |  |  |  |
| Yes | 19 (8.1%) | | 76.9% |  |  |  |
| Preoperative bowel perforation |  | |  | 0.317 |  |  |
| No | 229 (97.4%) | | 90.1% |  |  |  |
| Yes | 6 (2.6%) | | 75.0% |  |  |  |
| Clinical N stage |  | | | 0.083 |  |  |
| N0 | 69 (29.4%) | | 95.6% |  |  |  |
| N1/2 | 166 (70.6%) | | 87.5% |  |  |  |
| Tumor diameter, mm |  | | | 0.664 |  |  |
| <45 | 100 (42.6%) | 90.5% | |  |  |  |
| ≥45 | 135 (57.4%) | 89.3% | |  |  |  |
| Tumor differentiation |  | | | 0.498 |  |  |
| Well/mod | 210 (89.4%) | | 90.0% |  |  |  |
| Por/sig/muc | 24 (10.2%) | | 87.5% |  |  |  |
| Others | 1 (0.4%) | | NA |  |  |  |
| Depth of tumor invasion |  | | | 0.001 | 2.557 (1.083–6.039) | 0.032 |
| Pathological T3 | 190 (80.9%) | | 92.3% |  |  |  |
| Pathological T4 | 45 (19.1%) | | 79.4% |  |  |  |
| Number of harvested lymph node |  | | | 0.692 |  |  |
| <12 | 135 (57.4%) | | 89.5% |  |  |  |
| ≥12 | 100 (42.6%) | | 90.3% |  |  |  |
| Venous invasion |  | | | 0.590 |  |  |
| Negative | 93 (39.6%) | | 90.6% |  |  |  |
| Positive | 142 (60.4%) | | 89.3% |  |  |  |
| Lymphatic invasion |  | | | 0.302 |  |  |
| Negative | 108 (46.0%) | | 87.1% |  |  |  |
| Positive | 127 (54.0%) | | 92.2% |  |  |  |

WBC, white blood cell; CRP, C-reactive protein; HRs, hazard ratios; CI, confidence interval.

**SUPPLEMENTARY TABLE 4. Clinical characteristics of the patients with left-sided colon cancer in the non-enlarged lymph-node and enlarged lymph-node groups**

|  | *All (n = 188)* | *Non-enlarged lymph node (n = 71)* | *Enlarged lymph node (n = 117)* | *p* |
| --- | --- | --- | --- | --- |
| Age, median (IQR), years | 71 (64–78) | 74 (65–80.5) | 70 (63–77) | 0.048 |
| Sex |  |  |  | 0.548 |
| Male | 97 (51.6%) | 39 (54.9%) | 58 (49.6%) |  |
| Female | 91 (48.4%) | 32 (45.1%) | 59 (50.4%) |  |
| BMI, median (IQR), kg/m^2^ | 21.8 (19.8–23.9) | 21.8 (19.4–23.7) | 21.8 (20.1–24.1) | 0.439 |
| Smoking |  |  |  | 0.880 |
| No | 106 (56.4%) | 41 (57.7%) | 65 (55.6%) |  |
| Yes | 82 (43.6%) | 30 (42.3%) | 52 (44.4%) |  |
| ASA-PS score |  |  |  | 0.178 |
| I | 31 (16.5%) | 8 (11.3%) | 23 (19.7%) |  |
| II | 139 (73.9%) | 58 (81.7%) | 81 (69.2%) |  |
| III | 18 (9.6%) | 5 (7.0%) | 13 (11.1%) |  |
| IV | 0 (0%) | 0 (0%) | 0 (0%) |  |
| Allergy |  |  |  | 0.084 |
| No | 179 (95.2%) | 65 (91.5%) | 114 (97.4%) |  |
| Yes | 9 (4.8%) | 6 (8.5%) | 3 (2.6%) |  |
| Preoperative bowel obstruction |  |  |  | 0.769 |
| No | 175 (93.1%) | 67 (94.4%) | 108 (92.3%) |  |
| Yes | 13 (6.9%) | 4 (5.6%) | 9 (7.7%) |  |
| Preoperative bowel perforation |  |  |  | 0.299 |
| No | 184 (97.9%) | 71 (100%) | 113 (96.6%) |  |
| Yes | 4 (2.1%) | 0 (0%) | 4 (3.4%) |  |
| Preoperative WBC level, median (IQR), μ/L | 6300 (5300–7500) | 6100 (5150–7300) | 6300 (5300–7700) | 0.352 |
| Preoperative hemoglobin level, median (IQR), g/dL | 12.5 (10.6–13.7) | 12.7 (11.1–14.0) | 12.2 (10.5–13.5) | 0.066 |
| Preoperative albumin level, median (IQR), g/dL | 4.0 (3.5–4.3) | 4.1 (3.7–4.4) | 4.0 (3.5–4.3) | 0.256 |
| Preoperative HbA1c level, median (IQR), mmol/ mol | 5.8 (5.5–6.2) | 5.8 (5.4–6.4) | 5.8 (5.5–6.1) | 0.985 |
| Preoperative CRP level, median (IQR), mg/dL | 0.15 (0.05–0.5) | 0.07 (0.04–0.35) | 0.21 (0.06–0.66) | 0.008 |
| Preoperative CEA level, median (IQR), ng/dL | 4.3 (2.3–7.3) | 3.0 (1.9–4.8) | 2.9 (1.8–6.0) | 0.604 |
| Preoperative CA19-9 level, median (IQR), U/mL | 9 (5.3–19.2) | 4.2 (2.4–6.5) | 4.3 (2.3–8.9) | 0.432 |
| Approaches |  |  |  | 0.059 |
| Open surgery | 28 (14.9%) | 6 (8.5%) | 22 (18.8%) |  |
| Conventional laparoscopic surgery | 160 (85.1%) | 65 (91.5% | 95 (81.2%) |  |
| Robotic-assisted laparoscopic surgery | 0 (0%) | 0 (0%) | 0 (0%) |  |
| Operative time, median (IQR), min | 206.5 (171.8–254.3) | 192 (166.5–222.5) | 215 (175–270) | 0.009 |
| Blood loss, median (IQR), mL | 10 (5–50) | 5 (5–32.5) | 13 (5–78) | 0.024 |
| Tumor diameter, median (IQR), mm | 44 (33–55.5) | 35 (25.5–41.5) | 50 (40–60) | <0.001 |
| Tumor differentiation |  |  |  | 0.783 |
| Well/mod | 182 (96.8%) | 70 (98.6%) | 112 (95.7%) |  |
| Por/sig/muc | 5 (2.7%) | 1 (1.4%) | 4 (3.4%) |  |
| Others | 1 (0.5%) | 0 (0%) | 1 (0.9%) |  |
| Pathological T stage |  |  |  | 0.012 |
| T3 | 145 (77.1%) | 62 (87.3%) | 83 (70.9%) |  |
| T4 | 43 (22.9%) | 9 (12.7%) | 34 (29.1%) |  |
| Number of lymph nodes examined, median (IQR) | 19.5 (16.3–24) | 18 (13–24) | 23 (19–30) | <0.001 |
| Lymphatic invasion |  |  |  | 0.363 |
| Negative | 109 (58.0%) | 38 (53.5%) | 71 (60.7%) |  |
| Positive | 79 (42.0%) | 33 (46.5%) | 46 (39.3%) |  |
| Venous invasion |  |  |  | 0.344 |
| Negative | 64 (34.0%) | 21 (29.6%) | 43 (36.8%) |  |
| Positive | 124 (66.0%) | 50 (70.4%) | 74 (63.2%) |  |
| Adjuvant chemotherapy | 26 (13.8%) | 9 (12.7%) | 17 (14.5%) | 0.829 |
| Recurrence rate | 20 (10.6%) | 8 (11.3%) | 12 (10.3%) | 0.812 |
| Liver | 11 (5.9%) | 6 (8.5%) | 5 (4.3%) |  |
| Lung | 4 (2.1%) | 2 (2.8%) | 2 (1.7%) |  |
| Peritoneal dissemination | 6 (3.2%) | 1 (1.4%) | 5 (4.3%) |  |
| Local | 1 (0.5%) | 1 (1.4%) | 0 (0%) |  |
| Others | 5 (2.6%) | 3 (4.2%) | 2 (1.7%) |  |
| Median follow-up period (IQR), months | 63.4 (49.0–76.4) | 63.6 (49.2–73.0) | 63.1 (49–48.6) | 0.295 |

IQR, interquartile range; BMI, body mass index; ASA-PS, American Society of Anesthesiologists Physical Status Classification System; HTN, hypertension; WBC, white blood cell; HbA1c, Hemoglobin A1c; CRP, C-reactive protein.

**SUPPLEMENTARY TABLE 5. Univariate and multivariate analyses of 5-year overall survival after resection of left-sided colon cancer**

| *Variable* | *All (n =188)* | |  | *Univariate analysis*  *(log-rank)* | *Multivariate analysis*  *(Cox hazard)* | |
| --- | --- | --- | --- | --- | --- | --- |
|  | *No. of patients* | | *5-year OS* | *p-value* | *HRs (95% CI)* | *p-value* |
| Age, years |  | |  | 0.591 |  |  |
| <70 | 74 (39.4%) | | 89.5% |  |  |  |
| ≥70 | 114 (60.6%) | | 91.0% |  |  |  |
| Sex |  | |  | 0.218 |  |  |
| Male | 97 (51.6%) | | 87.5% |  |  |  |
| Female | 91 (48.4%) | | 93.7% |  |  |  |
| Preoperative WBC level, μ/L |  | |  | 0.208 |  |  |
| ≤8500 | 159 (84.6%) | | 88.7% |  |  |  |
| >8500 | 29 (15.4%) | | 100% |  |  |  |
| Preoperative hemoglobin level, g/dL |  | |  | 0.004 | 3.010 (1.243–7.288) | 0.015 |
| <12 | 81 (43.1%) | | 84.9% |  |  |  |
| ≥12 | 107 (56.9%) | | 93.9% |  |  |  |
| Preoperative albumin level, g/dL |  | |  | 0.019 | 1.500 (0.637–3.530) | 0.354 |
| <4.0 | 80 (42.6%) | | 85.1% |  |  |  |
| ≥4.0 | 108 (57.4%) | | 94.0% |  |  |  |
| Preoperative CRP level, mg/dL |  | |  | 0.148 |  |  |
| ≤0.3 | 121 (64.4%) | | 92.5% |  |  |  |
| >0.3 | 67 (35.6%) | | 86.4% |  |  |  |
| Preoperative CEA level, ng/dL |  | |  | 0.313 |  |  |
| ≤5.0 | 111 (59.0%) | | 91.8% |  |  |  |
| >5.0 | 77 (41.0%) | | 88.2% |  |  |  |
| Preoperative bowel obstruction |  | |  | 0.310 |  |  |
| No | 175 (93.1%) | | 91.1% |  |  |  |
| Yes | 13 (6.9%) | | 80.8% |  |  |  |
| Preoperative bowel perforation |  | |  | 0.570 |  |  |
| No | 184 (97.9%) | | 90.2% |  |  |  |
| Yes | 4 (2.1%) | | 100% |  |  |  |
| Clinical-N stage |  | | | 0.038 | 0.467 (0.209–1.043) | 0.063 |
| N0 | 71 (37.8%) | | 86.2% |  |  |  |
| N1/2 | 117 (62.2%) | | 92.9% |  |  |  |
| Tumor diameter, mm |  | | | 0.584 |  |  |
| <45 | 96 (51.1%) | 86.6% | |  |  |  |
| ≥45 | 92 (48.9%) | 81.9% | |  |  |  |
| Tumor differentiation |  | | | 0.656 |  |  |
| Well/mod | 182 (96.8%) | | 90.1% |  |  |  |
| Por/sig/muc | 5 (2.7%) | | 100% |  |  |  |
| Others | 1 (0.5%) | | NA |  |  |  |
| Depth of tumor invasion |  | | | 0.726 |  |  |
| Pathological T3 | 145 (77.1%) | | 90.7% |  |  |  |
| Pathological T4 | 43 (22.9%) | | 89.3% |  |  |  |
| Number of harvested lymph node |  | | | 0.004 | 2.899 (1.132–7.421) | 0.027 |
| <12 | 81 (43.1%) | | 84.9% |  |  |  |
| ≥12 | 107 (56.9%) | | 93.9% |  |  |  |
| Venous invasion |  | | | 0.657 |  |  |
| Negative | 64 (34.0%) | | 89.4% |  |  |  |
| Positive | 124 (66.0%) | | 91.0% |  |  |  |
| Lymphatic invasion |  | | | 0.043 | 1.636 (0.732–3.655) | 0.231 |
| Negative | 109 (58.0%) | | 93.8% |  |  |  |
| Positive | 79 (42.0%) | | 85.5% |  |  |  |
| Adjuvant chemotherapy |  | |  | 0.737 |  |  |
| No | 162 (86.2%) | | 90.9% |  |  |  |
| Yes | 26 (13.8%) | | 87.6% |  |  |  |

WBC, white blood cell; CRP, C-reactive protein; HRs, hazard ratios; CI, confidence interval.

**SUPPLEMENTARY TABLE 6. Univariate and multivariate analyses of 5-year relapse-free survival after resection of left-sided colon cancer**

| *Variable* | *All (n =188)* | |  | *Univariate analysis*  *(log-rank)* | *Multivariate analysis*  *(Cox hazard)* | |
| --- | --- | --- | --- | --- | --- | --- |
|  | *No. of patients* | | *5-year RFS* | *p-value* | *HRs (95% CI)* | *p-value* |
| Age, years |  | |  | 0.063 |  |  |
| <70 | 74 (39.4%) | | 83.6% |  |  |  |
| ≥70 | 114 (60.6%) | | 93.2% |  |  |  |
| Sex |  | |  | 0.886 |  |  |
| Male | 97 (51.6%) | | 90.1% |  |  |  |
| Female | 91 (48.4%) | | 88.5% |  |  |  |
| Preoperative WBC level, μ/L |  | |  | 0.183 |  |  |
| ≤8500 | 159 (84.6%) | | 87.4% |  |  |  |
| >8500 | 29 (15.4%) | | 100% |  |  |  |
| Preoperative hemoglobin level, g/dL |  | |  | 0.481 |  |  |
| <12 | 81 (43.1%) | | 89.1% |  |  |  |
| ≥12 | 107 (56.9%) | | 89.8% |  |  |  |
| Preoperative albumin level, g/dL |  | |  | 0.062 |  |  |
| <4.0 | 80 (42.6%) | | 83.9% |  |  |  |
| ≥4.0 | 108 (57.4%) | | 93.1% |  |  |  |
| Preoperative CRP level, mg/dL |  | |  | 0.846 |  |  |
| ≤0.3 | 121 (64.4%) | | 90.2% |  |  |  |
| >0.3 | 67 (35.6%) | | 87.5% |  |  |  |
| Preoperative CEA level, ng/dL |  | |  | 0.068 |  |  |
| ≤5.0 | 111 (59.0%) | | 85.9% |  |  |  |
| >5.0 | 77 (41.0%) | | 94.5% |  |  |  |
| Preoperative bowel obstruction |  | |  | 0.093 |  |  |
| No | 175 (93.1%) | | 90.6% |  |  |  |
| Yes | 13 (6.9%) | | 72.9% |  |  |  |
| Preoperative bowel perforation |  | |  | 0.476 |  |  |
| No | 184 (97.9%) | | 89.1% |  |  |  |
| Yes | 4 (2.1%) | | 100% |  |  |  |
| Clinical N stage |  | | | 0.629 |  |  |
| N0 | 71 (37.8%) | | 87.3% |  |  |  |
| N1/2 | 117 (62.2%) | | 90.5% |  |  |  |
| Tumor diameter, mm |  | | | 0.244 |  |  |
| <45 | 96 (51.1%) | 91.1% | |  |  |  |
| ≥45 | 92 (48.9%) | 87.5% | |  |  |  |
| Tumor differentiation |  | | | 0.601 |  |  |
| Well/mod | 182 (96.8%) | | 89.8% |  |  |  |
| Por/sig/muc | 5 (2.7%) | | 50.0% |  |  |  |
| Others | 1 (0.5%) | | NA |  |  |  |
| Depth of tumor invasion |  | | | 0.001 |  |  |
| Pathological T3 | 145 (77.1%) | | 94.9% |  |  |  |
| Pathological T4 | 43 (22.9%) | | 71.8% |  |  |  |
| Number of harvested lymph node |  | | | 0.481 |  |  |
| <12 | 81 (43.1%) | | 89.1% |  |  |  |
| ≥12 | 107 (56.9%) | | 89.8% |  |  |  |
| Venous invasion |  | | | 0.410 |  |  |
| Negative | 64 (34.6%) | | 84.0% |  |  |  |
| Positive | 124 (66.0%) | | 92.5% |  |  |  |
| Lymphatic invasion |  | | | 0.917 |  |  |
| Negative | 109 (58.0%) | | 88.6% |  |  |  |
| Positive | 79 (42.0%) | | 90.9% |  |  |  |

WBC, white blood cell; CRP, C-reactive protein; HRs, hazard ratios; CI, confidence interval.

**SUPPLEMENTARY TABLE 7. Clinical characteristics of rectal cancer patients in the non-enlarged lymph-node and enlarged lymph-node groups**

|  | *All (n = 139)* | *Non-enlarged lymph node (n = 45)* | *Enlarged lymph node (n = 94)* | *p* |
| --- | --- | --- | --- | --- |
| Age, median (IQR), years | 70 (64–77) | 73 (65–77) | 68.5 (64–77) | 0.414 |
| Sex |  |  |  | 0.454 |
| Male | 50 (36.0%) | 14 (31.1%) | 36 (38.3%) |  |
| Female | 89 (64.0%) | 31 (68.9%) | 58 (61.7%) |  |
| BMI, median (IQR), kg/m^2^ | 22.4 (20.8–24.9) | 22.1 (20.9–25.0) | 22.6 (20.6–24.6) | 0.892 |
| Smoking |  |  |  | 0.589 |
| No | 67 (48.2%) | 20 (44.4%) | 47 (50%) |  |
| Yes | 72 (51.8%) | 25 (55.6%) | 47 (50%) |  |
| ASA-PS score |  |  |  | 0.693 |
| I | 13 (9.4%) | 3 (6.7%) | 10 (10.6%) |  |
| II | 108 (77.7%) | 35 (77.8%) | 73 (77.7%) |  |
| III | 18 (12.9%) | 7 (15.6%) | 11 (11.7%) |  |
| IV | 0 (0%) | 0 (0%) | 0 (0%) |  |
| Allergy |  |  |  | 0.544 |
| No | 137 (98.6%) | 44 (97.8%) | 93 (98.9%) |  |
| Yes | 2 (1.4%) | 1 (2.2%) | 1 (1.1%) |  |
| Preoperative bowel obstruction |  |  |  | 1 |
| No | 138 (99.3%) | 45 (100%) | 93 (98.9%) |  |
| Yes | 1 (0.7%) | 0 (0%) | 1 (1.1%) |  |
| Preoperative bowel perforation |  |  |  | 1 |
| No | 134 (96.4%) | 44 (97.8%) | 90 (95.7%) |  |
| Yes | 5 (3.6%) | 1 (2.2%) | 4 (4.3%) |  |
| Preoperative WBC level, median (IQR), μ/L | 6200 (5100–7500) | 6900 (4800–7800) | 6250 (5200–7300) | 0.560 |
| Preoperative hemoglobin level, median (IQR), g/dL | 12.8 (11.5–13.8) | 13.1 (11.9–14.1) | 12.7 (11.3–13.6) | 0.091 |
| Preoperative albumin level, median (IQR), g/dL | 4.1 (3.8–4.3) | 4.2 (3.9–4.5) | 4.0 (3.8–4.3) | 0.042 |
| Preoperative HbA1c level, median (IQR), mmol/ mol | 5.8 (5.5–6.3) | 5.9 (5.5–6.4) | 5.8 (5.5–6.3) | 0.856 |
| Preoperative CRP level, median (IQR), mg/dL | 0.10 (0.04–0.22) | 0.08 (0.03–0.14) | 0.10 (0.05–0.23) | 0.291 |
| Preoperative CEA level, median (IQR), ng/dL | 3.8 (2.3–6.8) | 3.3 (2.3–6.0) | 4.4 (2.3–8.1) | 0.145 |
| Preoperative CA19-9 level, median (IQR), U/mL | 11.1 (6.2–19.9) | 12.1 (6.3–20) | 10.6 (6.0–19) | 0.684 |
| Approaches |  |  |  | 0.776 |
| Open surgery | 19 (13.7%) | 5 (11.1%) | 14 (14.9%) |  |
| Conventional laparoscopic surgery | 107 (77.0%) | 35 (77.8%) | 72 (76.6%) |  |
| Robotic-assisted laparoscopic surgery | 13 (9.4%) | 5 (11.1%) | 8 (8.5%) |  |
| Operative time, median (IQR), min | 263 (218–350) | 260 (217–305) | 264 (218.5–379.3) | 0.288 |
| Blood loss, median (IQR), mL | 20 (5–114.5) | 20 (5–80) | 18.5 (5–137.3) | 0.960 |
| Tumor diameter, median (IQR), mm | 50 (37.5–60) | 35 (32–49) | 55 (42–65) | <0.001 |
| Tumor differentiation |  |  |  | 1 |
| Well/mod | 135 (97.1%) | 44 (97.8%) | 91 (96.8%) |  |
| Por/sig/muc | 4 (2.9%) | 1 (2.2%) | 3 (3.2%) |  |
| Others | 0 (%) | 0 (0%) | 0 (%) |  |
| Pathological T stage |  |  |  | 0.142 |
| T3 | 116 (83.5%) | 41 (91.1%) | 75 (79.8%) |  |
| T4 | 23 (16.5%) | 4 (8.9%) | 19 (20.2%) |  |
| Number of lymph nodes examined, median (IQR) | 12 (8–18) | 16 (10–21) | 21 (16–31.8) | <0.001 |
| Lymphatic invasion |  |  |  | 1 |
| Negative | 68 (48.9%) | 22 (48.9%) | 46 (48.9%) |  |
| Positive | 71 (51.1%) | 23 (51.1%) | 48 (51.1%) |  |
| Venous invasion |  |  |  | 0.703 |
| Negative | 46 (33.1%) | 16 (35.6%) | 30 (31.9%) |  |
| Positive | 93 (66.9%) | 29 (64.4%) | 64 (68.1%) |  |
| Adjuvant chemotherapy | 26 (18.7%) | 5 (11.1%) | 21 (22.3%) | 0.162 |
| Recurrence rate | 17 (12.2%) | 8 (17.8%) | 9 (9.6%) | 0.177 |
| Liver | 8 (5.8%) | 4 (8.9%) | 4 (4.3%) |  |
| Lung | 4 (2.9%) | 2 (4.4%) | 2 (2.1%) |  |
| Peritoneal dissemination | 4 (2.9%) | 1 (2.2%) | 3 (3.2%) |  |
| Local | 2 (1.4%) | 0 (0%) | 2 (2.1%) |  |
| Others | 0 (0%) | 0 (0%) | 0 (0%) |  |
| Median follow-up period (IQR), months | 62.1 (41.2–77.9) | 60.9 (30.8–74.9) | 63.8 (57.5–78.3) | 0.060 |

IQR, interquartile range; BMI, body mass index; ASA-PS, American Society of Anesthesiologists Physical Status Classification System; HTN, hypertension; WBC, white blood cell; HbA1c, Hemoglobin A1c; CRP, C-reactive protein.

**SUPPLEMENTARY TABLE 8. Univariate and multivariate analyses of 5-year overall survival after resection of rectal cancer**

| *Variable* | *All (n =139)* | |  | *Univariate analysis*  *(log-rank)* | *Multivariate analysis*  *(Cox hazard)* | |
| --- | --- | --- | --- | --- | --- | --- |
|  | *No. of patients* | | *5-year OS* | *p-value* | *HRs (95% CI)* | *p-value* |
| Age, years |  | |  | 0.004 | 4.530 (1.256–16.34) | 0.021 |
| <70 | 67 (48.2%) | | 96.8% |  |  |  |
| ≥70 | 72 (51.8%) | | 83.9% |  |  |  |
| Sex |  | |  | 0.655 |  |  |
| Male | 89 (64.0%) | | 90.2% |  |  |  |
| Female | 50 (36.0%) | | 89.2% |  |  |  |
| Preoperative WBC level, μ/L |  | |  | 0.934 |  |  |
| ≤8500 | 128 (92.1%) | | 89.8% |  |  |  |
| >8500 | 11 (7.9%) | | 90.0% |  |  |  |
| Preoperative hemoglobin level, g/dL |  | |  | 0.026 | 2.251 (0.811–6.247) | 0.119 |
| <12 | 92 (66.2%) | | 95.1% |  |  |  |
| ≥12 | 47 (33.8%) | | 79.3% |  |  |  |
| Preoperative albumin level, g/dL |  | |  | 0.200 |  |  |
| <4.0 | 55 (39.6%) | | 86.6% |  |  |  |
| ≥4.0 | 84 (60.4%) | | 92.1% |  |  |  |
| Preoperative CRP level, mg/dL |  | |  | 0.909 |  |  |
| ≤0.3 | 111 (79.9%) | | 89.1% |  |  |  |
| >0.3 | 28 (20.1%) | | 92.6% |  |  |  |
| Preoperative CEA level, ng/dL |  | |  | 0.573 |  |  |
| ≤5.0 | 88 (63.3%) | | 89.8% |  |  |  |
| >5.0 | 51 (36.7%) | | 89.6% |  |  |  |
| Preoperative bowel obstruction |  | |  | <0.001 | 27.07 (2.345–31.26) | 0.008 |
| No | 138 (99.3%) | | 90.5% |  |  |  |
| Yes | 1 (0.7%) | | NA |  |  |  |
| Preoperative bowel perforation |  | |  | 0.528 |  |  |
| No | 134 (96.4%) | | 90.2% |  |  |  |
| Yes | 5 (3.6%) | | 80.0% |  |  |  |
| Clinical-N stage |  | | | 0.069 |  |  |
| N0 | 45 (32.4%) | | 80.3% |  |  |  |
| N1/2 | 94 (67.6%) | | 94.2% |  |  |  |
| Tumor diameter, mm |  | | | 0.489 |  |  |
| <45 | 54 (38.8%) | 90.2% | |  |  |  |
| ≥45 | 85 (61.2%) | 89.5% | |  |  |  |
| Tumor differentiation |  | | | 0.532 |  |  |
| Well/mod | 135 (97.1%) | | 89.5% |  |  |  |
| Por/sig/muc | 4 (2.9%) | | 100% |  |  |  |
| Others | 0 (0%) | | - |  |  |  |
| Depth of tumor invasion |  | | | 0.137 |  |  |
| Pathological T3 | 116 (83.5%) | | 90.4% |  |  |  |
| Pathological T4 | 23 (16.5%) | | 86.5% |  |  |  |
| Number of harvested lymph node |  | | | 0.754 |  |  |
| <12 | 26 (18.7%) | | 86.6% |  |  |  |
| ≥12 | 113 (81.3%) | | 90.5% |  |  |  |
| Venous invasion |  | | | 0.393 |  |  |
| Negative | 46 (33.1%) | | 90.2% |  |  |  |
| Positive | 93 (66.9%) | | 89.5% |  |  |  |
| Lymphatic invasion |  | | | 0.892 |  |  |
| Negative | 68 (48.9%) | | 89.0% |  |  |  |
| Positive | 71 (51.1%) | | 90.8% |  |  |  |
| Adjuvant chemotherapy |  | |  | 0.426 |  |  |
| No | 113 (81.3%) | | 89.2% |  |  |  |
| Yes | 26 (18.7%) | | 92.0% |  |  |  |

WBC, white blood cell; CRP, C-reactive protein; HRs, hazard ratios; CI, confidence interval.

**SUPPLEMENTARY TABLE 9. Univariate and multivariate analyses of 5-year relapse-free survival after resection of rectal cancer**

| *Variable* | *All (n =139)* | |  | *Univariate analysis*  *(log-rank)* | *Multivariate analysis*  *(Cox hazard)* | |
| --- | --- | --- | --- | --- | --- | --- |
|  | *No. of patients* | | *5-year RFS* | *p-value* | *HRs (95% CI)* | *p-value* |
| Age, years |  | |  | 0.586 |  |  |
| <70 | 67 (48.2%) | | 90.4% |  |  |  |
| ≥70 | 72 (51.8%) | | 89.4% |  |  |  |
| Sex |  | |  | 0.437 |  |  |
| Male | 50 (36.0%) | | 93.5% |  |  |  |
| Female | 89 (64.0%) | | 87.6% |  |  |  |
| Preoperative WBC level, μ/L |  | |  | 0.232 |  |  |
| ≤8500 | 128 (92.1%) | | 90.5% |  |  |  |
| >8500 | 11 (7.9%) | | 81.8% |  |  |  |
| Preoperative hemoglobin level, g/dL |  | |  | 0.257 |  |  |
| <12 | 92 (66.2%) | | 92.0% |  |  |  |
| ≥12 | 47 (33.8%) | | 85.1% |  |  |  |
| Preoperative albumin level, g/dL |  | |  | 0.690 |  |  |
| <4.0 | 84 (60.4%) | | 89.7% |  |  |  |
| ≥4.0 | 55 (39.6%) | | 90.3% |  |  |  |
| Preoperative CRP level, mg/dL |  | |  | 0.622 |  |  |
| ≤0.3 | 111 (79.9%) | | 91.2% |  |  |  |
| >0.3 | 28 (20.1%) | | 84.0% |  |  |  |
| Preoperative CEA level, ng/dL |  | |  | 0.394 |  |  |
| ≤5.0 | 88 (63.3%) | | 91.6% |  |  |  |
| >5.0 | 51 (36.7%) | | 86.8% |  |  |  |
| Preoperative bowel obstruction |  | |  | 0.932 |  |  |
| No | 138 (99.3%) | | 89.8% |  |  |  |
| Yes | 1 (0.7%) | | NA |  |  |  |
| Preoperative bowel perforation |  | |  | 0.587 |  |  |
| No | 134 (96.4%) | | 90.1% |  |  |  |
| Yes | 5 (3.6%) | | 80.0% |  |  |  |
| Clinical N stage |  | | | 0.158 |  |  |
| N0 | 45 (32.4%) | | 83.1% |  |  |  |
| N1/2 | 94 (67.6%) | | 93.1% |  |  |  |
| Tumor diameter, mm |  | | | 0.096 |  |  |
| <45 | 54 (38.8%) | 96.2% | |  |  |  |
| ≥45 | 85 (61.2%) | 86.0% | |  |  |  |
| Tumor differentiation |  | | | 0.522 |  |  |
| Well/mod | 135 (97.1%) | | 89.5% |  |  |  |
| Por/sig/muc | 4 (2.9%) | | 100% |  |  |  |
| Others | 0 (0%) | | NA |  |  |  |
| Depth of tumor invasion |  | | | 0.029 |  |  |
| Pathological T3 | 116 (83.5%) | | 92.6% |  |  |  |
| Pathological T4 | 23 (16.5%) | | 76.4% |  |  |  |
| Number of harvested lymph node |  | | | 0.280 |  |  |
| <12 | 26 (18.7%) | | 83.0% |  |  |  |
| ≥12 | 113 (81.3%) | | 91.3% |  |  |  |
| Venous invasion |  | | | 0.375 |  |  |
| Negative | 46 (33.1%) | | 89.9% |  |  |  |
| Positive | 93 (66.9%) | | 89.7% |  |  |  |
| Lymphatic invasion |  | | | 0.231 |  |  |
| Negative | 68 (48.9%) | | 88.7% |  |  |  |
| Positive | 71 (51.1%) | | 90.9% |  |  |  |

WBC, white blood cell; CRP, C-reactive protein; HRs, hazard ratios; CI, confidence interval.
